# Giant anomalous Nernst signal in the antiferromagnet YbMnBi_2_

**DOI:** 10.1038/s41563-021-01149-2

**Published:** 2021-11-22

**Authors:** Yu Pan, Congcong Le, Bin He, Sarah J. Watzman, Mengyu Yao, Johannes Gooth, Joseph P. Heremans, Yan Sun, Claudia Felser

**Affiliations:** 1 Max Planck Institute for Chemical Physics of Solids, Dresden, Germany; 2 Department of Mechanical and Aerospace Engineering, The Ohio State University, Columbus, OH USA; 3 Department of Mechanical and Materials Engineering, University of Cincinnati, Cincinnati, OH USA; 4 Department of Materials Science and Engineering, The Ohio State University, Columbus, OH USA; 5 Department of Physics, The Ohio State University, Columbus, OH USA

**Keywords:** Electronic properties and materials, Thermoelectrics

## Abstract

A large anomalous Nernst effect (ANE) is crucial for thermoelectric energy conversion applications because the associated unique transverse geometry facilitates module fabrication. Topological ferromagnets with large Berry curvatures show large ANEs; however, they face drawbacks such as strong magnetic disturbances and low mobility due to high magnetization. Herein, we demonstrate that YbMnBi_2_, a canted antiferromagnet, has a large ANE conductivity of ~10 A m^−1^ K^−1^ that surpasses large values observed in other ferromagnets (3–5 A m^−1^ K^−1^). The canted spin structure of Mn guarantees a non-zero Berry curvature, but generates only a weak magnetization three orders of magnitude lower than that of general ferromagnets. The heavy Bi with a large spin–orbit coupling enables a large ANE and low thermal conductivity, whereas its highly dispersive *p*_*x*/*y*_ orbitals ensure low resistivity. The high anomalous transverse thermoelectric performance and extremely small magnetization make YbMnBi_2_ an excellent candidate for transverse thermoelectrics.

## Abstract (web summary)

The anomalous Nernst effect (ANE) in topological materials with large Berry curvature shows great potential for transverse thermoelectrics, but antiferromagnets typically show small ANEs. The antiferromagnet YbMnBi_2_ has an ANE thermopower of 3 μV K^−1^, similar to ferromagnets, and a larger ANE conductivity.

## Main

Topological electronic structures lay the foundation for new functionalities and are crucial for various applications, including thermoelectric energy conversion^1–3^. In the past few decades, many good longitudinal thermoelectric materials have been demonstrated to be topological insulators^4,5^. Recently, with the emergence of topological semimetals, the anomalous Nernst effect (ANE) has attracted increasing attention for transverse thermoelectric applications^2,3,6–10^. Owing to the large Berry curvature near the Fermi energy, large-ANE thermopowers have been achieved in a few topological ferromagnets^2,3,6–12^, which show great potential for stable and precise temperature control, particularly in micro- or nanosized devices^13,14^. For example, Co_3_Sn_2_S_2_ (refs. ^7,8^), Co_2_MnGa (refs. ^3,9^) and Fe_3_Ga (ref. ^2^) showed ANE thermopowers of 3–8 μV K^−1^ and ANE conductivities of 0.5–5 A m^−1^ K^−1^, and recently UCo_0.8_Ru_0.2_Al (ref. ^15^) was reported to have a colossal ANE thermopower of 23 μV K^−1^ and a large ANE conductivity of 15 A m^−1^ K^−1^. So far, large ANEs have been reported in only a few topological ferromagnets, and topological noncollinear antiferromagnets have rarely been studied, except for Mn_3_*X* (*X* = Sn and Ge)^6,10,12^. The search for large ANEs in noncollinear topological antiferromagnets will yield advantages by broadening the material platform for transverse thermoelectrics, as well as revealing novel topological phenomena.

To use the ANE for practical applications, low resistivity and thermal conductivity are also required. Moreover, at the device level, low magnetization and a small inherent stray field are important to eliminate magnetic disturbances and stabilize the remanent magnetization in the in-plane direction if using in a thin-film case^16^. From these viewpoints, ANE efficiency is coupled with various parameters, similar to the longitudinal Seebeck effect^17^, and additional magnetization makes the coupling more complicated. For ferromagnets in general, although strong ferromagnetism breaks time-reversal symmetry and offers a large Berry curvature, it also has some drawbacks. With local moment and itinerant electrons (usually due to the heavy *d* or even *f* bands) at the Fermi energy (*E*_F_), these ferromagnets mostly have relatively low mobility and high resistivity. In addition, the high magnetization and strong inherent stray field of ferromagnets introduce a strong magnetic disturbance, which causes interference with other electronic devices in practical applications.

Herein, considering the challenges outlined above, we highlight the importance of searching for materials with broken time-reversal symmetry and *p* bands or *p*–*d* hybridization near *E*_F_. YbMnBi_2_, as a canted antiferromagnet, can simultaneously realize broken time-reversal symmetry and relatively low resistivity with light band conduction. The canted spin structure at Mn sites breaks time-reversal symmetry. Meanwhile, low resistivity can be achieved owing to the sharp dispersion of the *p*_*x*/*y*_ orbitals of Bi. Moreover, the large spin–orbit coupling (SOC) from Bi is critical for band topology, transverse transport response and low thermal conductivity. Furthermore, the YbMnBi_2_ canted antiferromagnet has much smaller magnetization and inherent stray fields than other ferromagnets. All of these features demonstrate that YbMnBi_2_ could provide a new material platform beyond ferromagnets for transverse thermoelectric applications.

## Canted antiferromagnets for ANE thermoelectric applications

**Fig. 1:**
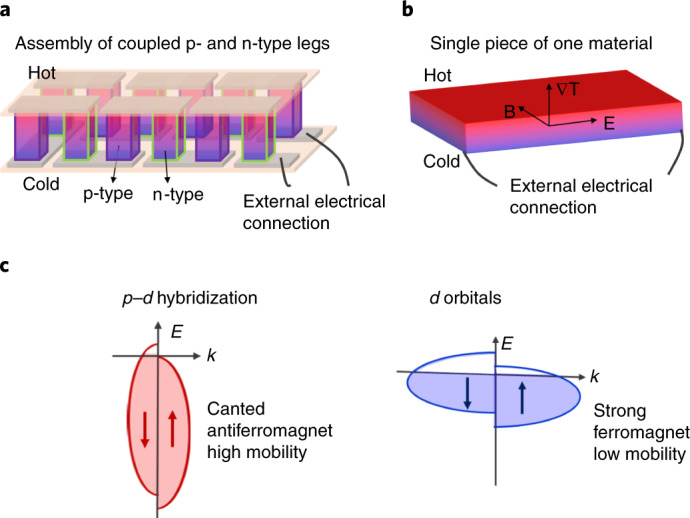
Illustration of thermoelectric devices and band dispersion. **a**,**b**, Schematics of thermoelectric devices based on the Seebeck effect (**a**) and Nernst effect (**b**). The Nernst device needs only one material and eliminates the complex electrical connections required in the Seebeck device, especially at the hot side. B, magnetic field; E, electrical field; ∇T, temperature gradient. **c**, Schematics of the band structure near *E*_F_ for canted YbMnBi_2_ (left) and general ferromagnets (right). YbMnBi_2_ shows a much sharper band dispersion and a higher mobility than general ferromagnets.

A Nernst device can greatly simplify module fabrication via its unique transverse geometry: the device can be made simply from one material, rather than needing both polarities of charge carriers in separate materials, as is required of Seebeck modules. As schematically shown in Fig. 1a, longitudinal Seebeck thermoelectric devices require coupled p- and n-type legs and assembled pairs^17,18^. In contrast, the Nernst device (Fig. 1b) offers various advantages that simplify module assembly. First, complex electrical connections and associated electrical resistances are eliminated because it requires only one material. Second, the voltage in Nernst devices can be probed in an isothermal plane, and the electrodes can be constructed at only the isothermal cold end; hence, such devices do not require contacts that are stable at high temperatures. Third, the output of a Nernst device scales extrinsically with the device size: manufacturing longer and thicker devices can increase the voltage output and temperature gradient, respectively. Increasing the size in either dimension would increase the associated potential drop.

YbMnBi_2_ outshines common ferromagnets for applications as a Nernst device in two ways: high mobility and low magnetization. First, as schematically shown in Fig. 1c, the bands of YbMnBi_2_ at *E*_F_ are very light in the *ab* plane, formed by the hybridization of *p* and *d* orbitals and thus guaranteeing a high mobility; they are usually heavy *d* or *f* orbitals for general ferromagnets. Second, the ferromagnetism originating from the canted spin structure of Mn is extremely weak. These two advantages are useful for thermoelectric applications owing to the low resistivity at the material level and small magnetic disturbance at the device level.

## The search for canted antiferromagnets

We have reviewed the magnetic topological materials identified recently by high-throughput calculations^19^. To introduce a canted spin structure, we examined the manganese pnictides *R*MnPn_2_, where *R* is a rare- or alkaline-earth metal, and Pn is a pnictide (Pn = P, As, Sb or Bi). A few canted manganese pnictides—such as SrMnSb_2_ (ref. ^20^), YbMnBi_2_ (ref. ^21^) and Ca_1−*x*_Na_*x*_MnBi_2_ (ref. ^22^)—are reported to be magnetic topological materials with a canted spin structure; they are therefore able to hold a non-zero Berry curvature^23,24^. Moreover, the bands contributed by pnictides are highly dispersive^25^, which can provide a low resistivity, especially compared with that of ferromagnets.

**Fig. 2:**
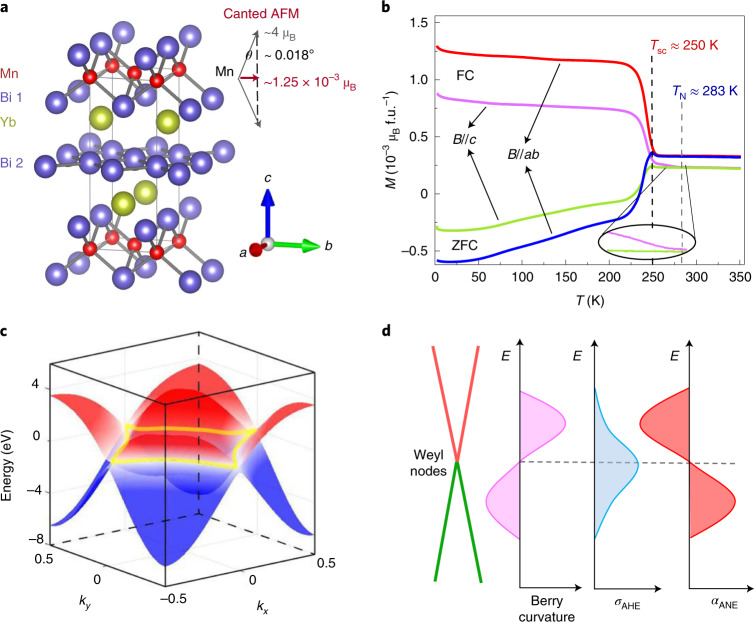
Canted spin structure and non-zero Berry curvature. **a**, Crystal structure of YbMnBi_2_. The spin structure is canted at Mn sites, contributing to weak ferromagnetism in the *ab* plane. **b**, Temperature dependence of magnetization *M* in the *ab* plane (magnetic field *B* parallel to *ab* plane, *B*//*ab*) and *c* axis (*B* parallel to *c* axis, *B*//*c*). Red and magenta curves denote field cooling (FC) under 1 T, whereas blue and green curves denote zero-field cooling (ZFC). The field for both FC and ZFC measurements is 100 Oe. A sharp increase in FC curves upon cooling indicates a spin canting temperature (*T*_sc_) of ~250 K; the Néel temperature (*T*_N_) is ~283 K. An extremely small *θ* of ~0.018° is resolved from the magnetization curves. **c**, The nodal line (yellow) formed near *E*_F_ in the *k*_*x*_*k*_*y*_ plane is shown (*k*_*x*_ and *k*_*y*_ are the *x* and *y* direction in the reciprocal space). The conduction and valence bands are shown in red and blue, respectively. **d**, Schematic of the *E*_F_ dependence of the Berry curvature (left), *σ*_AHE_ (middle) and *α*_ANE_ (right). The strength of the Berry curvature depends on *E*_F_, resulting in the maximum *σ*_AHE_ when *E*_F_ is near the Weyl nodes, whereas the maximum *α*_ANE_ lies at a different energy.

Given that Yb is relatively stable, Mn has a canted spin structure and Bi can induce large SOC, YbMnBi_2_ was investigated. As shown in Fig. 2a, YbMnBi_2_ crystallizes in a *P*4/*nmm* structure with two types of Bi: Bi 1 is bonded with Mn and Bi 2 forms an interlayer. The spin of Mn is antiferromagnetic along the *c* axis but canted in the *ab* plane. The Néel temperature was reported to be ~290 K (ref. ^26^), which is very close to that shown in Fig. 2b (~283 K), as also indicated by the turning point in the temperature-dependent resistivity (Supplementary Fig. 1). A clear ferromagnetic transition is observed, as shown in Fig. 2b. The sharp increase in magnetization upon cooling observed in the field cooling curves indicates a spin canting temperature of ~250 K. The field dependence of the magnetization is shown in Supplementary Fig. 2. Below 250 K, the saturation magnetization value is ~1.25 × 10^−3^ μ_B_ per formula unit (f.u.) in the *ab* plane, suggesting a very small canting angle (*θ*) of approximately 0.018°. Although the spin canting is very weak and may even be inaccessible in experiments requiring a strong signal^26,27^, it plays a crucial role in the anomalous thermal/electrical transport by inducing a non-zero Berry curvature.

Owing to the canted spin structure, YbMnBi_2_ has unique topological properties. When the SOC is ignored, a nodal line protected by glide-plane symmetry exists in the Brillouin zone because of the band inversion of the *p*_*x*/*y*_ orbitals of Bi 2, as shown by the yellow line in Fig. 2c. When SOC is included, the nodal line is gapped, resulting in two pairs of Weyl nodes and a non-zero Berry curvature. The strength of the Berry curvature depends on *E*_F_, and reaches its maximum when *E*_F_ is near the Weyl nodes. As schematically illustrated in Fig. 2d, the shift in *E*_F_ would probe the strength of the Berry curvature, therefore leading to the varying strength of the anomalous Hall effect (AHE) conductivity (*σ*_AHE_) and the ANE conductivity (*α*_ANE_)^28–30^. In this case, it is essential to optimize *E*_F_ to observe a large *σ*_AHE_ or *α*_ANE_, which are usually related to the quality of the single crystal.

According to theory, under a spin canting direction of (110)^21^, the magnetic point group of YbMnBi_2_ becomes *m*′*m*2′, and only transverse transport signals in the *ac*/*bc* and *ca*/*cb* configurations can be observed owing to mirror symmetry^31^. Taking the *cb* configuration as an example, this implies that the transverse signal is measured along the *c* axis while a temperature gradient is applied along the *b* axis and a magnetic field along the *a* axis. Experimental results are highly consistent with theoretical predictions: both the ANE and AHE are observed in the *bc* and *cb* configurations, but not in the *ab* plane (Supplementary Figs. 3 and 4). The non-zero Berry curvature is thus believed to be an essential cause of the ANE/AHE.

## Anomalous Nernst thermopower and anomalous Hall resistivity

**Fig. 3:**
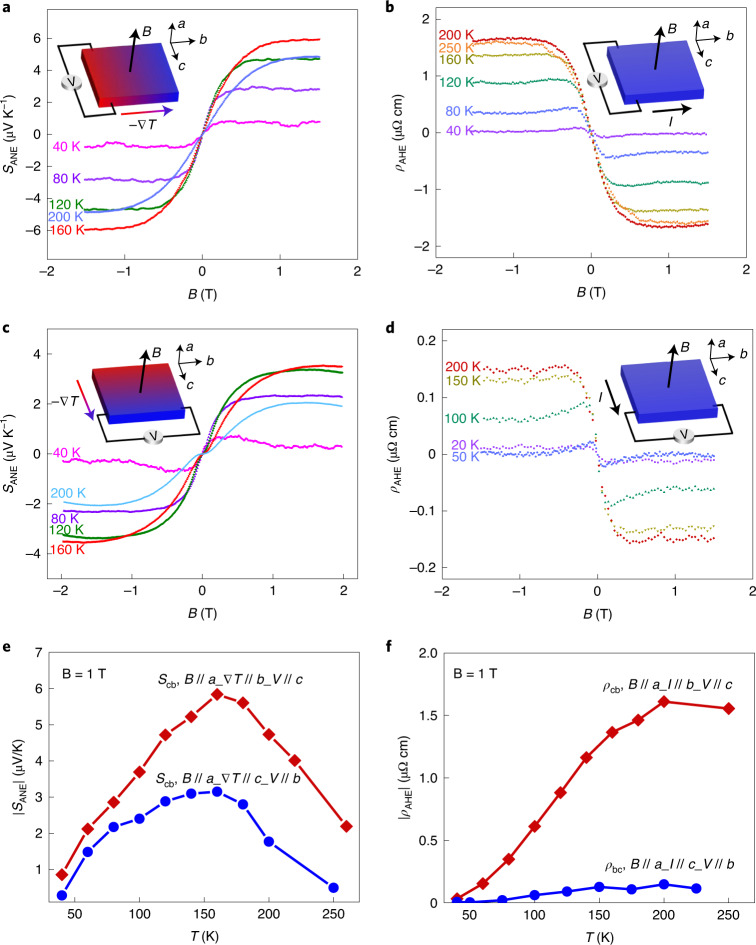
ANE thermopower and AHE resistivity. **a**–**d**, The magnetic field dependence of *S*_ANE_ (**a**,**c**) and *ρ*_AHE_ (**b**,**d**) in *cb* (*V*//*c*) for **a** and **b**, and *bc* (*V*//*b*) for **c** and **d** for the temperatures indicated. Herein, *cb* indicates the measurement of the Nernst/Hall voltage along the *c* axis and application of the temperature gradient along the *b* axis, and vice versa for *bc*; the magnetic field *B* is always along the *a* axis. The configuration of *B*, ∇*T*/*I* and *V* is schematically shown in the insets. *I*, current; *V*, voltage. **e**,**f**, The temperature dependence of the absolute values of *S*_ANE_ (**e**) and *ρ*_AHE_ (**f**), respectively, is shown at *B* = 1 T from 40 to 250 K. Directions of *B*, ∇*T*/*I* and *V* are parallel to the *a*, *b* and *c* axis, respectively, for *cb* and *bc*, as indicated by the notation in the figure.

The ANE thermopower (*S*_ANE_) and AHE resistivity (*ρ*_AHE_) (with Gerlach’s sign convention, as illustrated in Supplementary Fig. 5) in the *cb* and *bc* configurations show qualitatively identical behaviour but different values. *S*_ANE_ and *ρ*_AHE_ in both the *cb* (Fig. 3a,b) and *bc* (Fig. 3c,d) configurations saturate at ~1 T, which is relatively small, comparable to the saturation field of Co_2_MnGa^3,9^, and much lower than Fe_3_Ga (~2 T)^2^. *S*_ANE_ first increases and then decreases with heating, showing a peak at ~160 K (Fig. 3e); in contrast, *ρ*_AHE_ increases up to ~200 K (Fig. 3f). The maximum *S*_ANE_ values are ~6 µV K^−1^ and ~3 µV K^−1^ at 160 K in the *cb* and *bc* configurations, respectively, which is relatively large, especially for an antiferromagnet. This is remarkable, as the largest ANE signals reported so far in the antiferromagnets observed were ~0.5 µV K^−1^ in Mn_3_Sn (refs. ^6,12^) and ~1.2 µV K^−1^ in Mn_3_Ge (refs. ^10,32^).

## Intrinsic and extrinsic effects on ANE/AHE conductivity

**Fig. 4:**
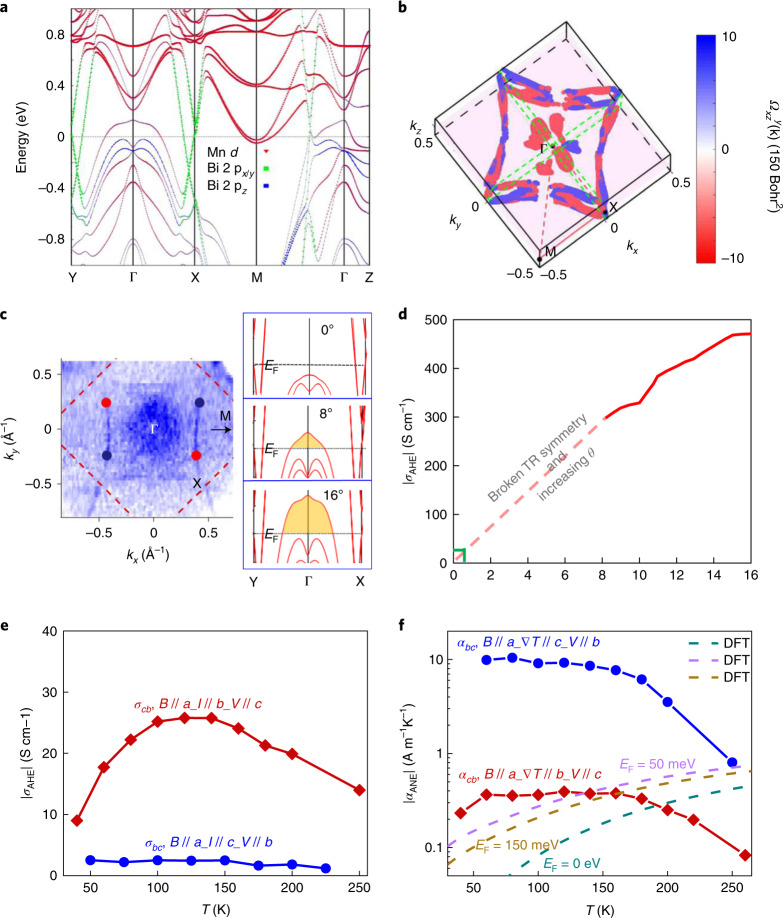
Theoretical analysis of ANE and AHE conductivity. **a**,**b**, Band structure of YbMnBi_2_ (**a**) (in which the different coloured lines denote the contribution from different orbitals from Mn and Bi elements) and Berry curvature distribution (**b**) in the Brillouin zone calculated from first principles by assuming *θ* = 10°. The total Berry curvature contribution from the electron pockets is negative. With a larger spin *θ*, the *d* orbitals of Mn join the conduction, and an additional negative Berry curvature is generated at the Г point. **c**, Left: ARPES intensity at *E*_F_ as a function of the *ab* plane wavevectors (*k*_*x*_ and *k*_*y*_) measured with 55 eV photons at *T* = 19 K. The ARPES intensity is integrated over the energy range of +10 meV to −10 meV with respect to *E*_F_. The inset shows two pairs of Weyl nodes. Right: the calculated band variation at the Г point with increasing *θ*. **d**, Calculated |*σ*_AHE_| as a function of *θ*. The solid line shows the calculated results, and the dashed line shows a linear extrapolation. **e**,**f**, Experimental results for the temperature dependence of |*σ*_AHE_| (**e**) and |*α*_ANE_| (**f**) in *cb* (*V*//*c*) and *bc* (*V*//*b*). Calculated |*α*_ANE_| values at different *E*_F_ are shown by the dashed lines in **f**.

To quantitatively understand the origin of the ANE and AHE in YbMnBi_2_, *α*_ANE_ and *σ*_AHE_ were analysed with first-principles calculations assuming *θ* = 10°. As *α*_ANE_ is an energy derivative of *σ*_AHE_ (ref. ^24^), we first investigated *σ*_AHE_, followed by *α*_ANE_. Two characteristics of the band structure are essential to understand the AHE and ANE. First, the hole pocket near the Г point, which stems from the hybridization of the *p*_*z*_ orbitals of Bi 1 and the *d* orbitals of Mn, should be far below *E*_F_ if *θ* is zero (Supplementary Fig. 6), but above *E*_F_ if *θ* is non-zero (Fig. 4a). The hole pocket near the Г point can generate a negative Berry curvature^31^, which enhances the total Berry curvature as the electron pockets (along Г–M, Г–X and Г–Y, originating from the *p*_x/y_ orbitals of Bi 2) also generate a negative Berry curvature (Fig. 4b). As shown in Fig. 4c, the Fermi surface at the Г point is observed in our crystal by angle-resolved photoemission spectroscopy (ARPES), which is highly important as it demonstrates the non-zero *θ* and additional negative Berry curvature from the Г band. The larger *θ* is, the more contribution to band conduction generated at the Г point, as illustrated on the right side of Fig. 4c and Supplementary Fig. 7. Figure 4d plots |*σ*_AHE_| as a function of *θ*; the calculated |*σ*_AHE_| has a nearly linear dependence on *θ* from 8° to 16°. As *σ*_AHE_ is 0 if *θ* = 0°, we extrapolated *σ*_AHE_ to 0 in the simplest way (with a linear dotted line), which therefore allowed us to roughly assess *σ*_AHE_ at extremely small *θ*.

Second, as the electronic bands of YbMnBi_2_ are strongly anisotropic and highly dispersive in the *ab* plane (Supplementary Fig. 8), there can be a large shift in *E*_F_. In fact, such a shift was observed in the Hall resistivity (Supplementary Fig. 4c): a transition from n-type to p-type was observed from 50 to 100 K. Such high anisotropy was also observed in the Seebeck coefficient (Supplementary Fig. 9), where positive and negative values coexisted in different crystallographic directions—this may indicate that YbMnBi_2_ is a goniopolar material^33^. This strong anisotropy makes it challenging to determine the actual *E*_F_ in specific directions as it can vary in different directions. On the basis of the analysis above, we attained a better understanding of |*σ*_AHE_| and |*α*_ANE_|.

As shown in Fig. 4e, the maximum experimental |*σ*_AHE_| can be matched with the theoretically predicted result with a very small *θ* (as illustrated by the point indicated with green lines in Fig. 4d). The decreasing trend above ~150 K can be attributed to the temperature disturbance of the spin structure; however, the increasing trend below 150 K is atypical. |*α*_ANE_| also shows an unusual decreasing trend, as shown in Fig. 4f. In addition, anisotropic behaviours are observed in *σ*_AHE_ and *α*_ANE_. Akgoz and Saunders^34,35^ combined the reciprocity relations with the crystal symmetries to derive the allowed symmetry operations in various point groups. With the magnetic point group of YbMnBi_2_ being *m*′*m2*′, one would expect that the Hall resistivity *ρ*_*bc*_(*H*_a_) = −*ρ*_*cb*_(−*H*_a_) (here, *ρ*_*bc*_ and *ρ*_*cb*_ are the Hall resistivity in *bc* and *cb*, respectively, and *H*_a_ is the external field along the *a* axis)^34^ and the same relation would be expected to hold for the conductivity tensors. The fact that this is not observed in Fig. 4e points to the absence of time-reversal symmetry and the anomalous origin of the Hall conductivities. For the thermoelectric conductivity tensor, the observation that *S*_*bc*_(*H*_a_) ≠ −*S*_*cb*_(−*H*_a_) (Fig. 3e) and *α*_*bc*_(*H*_a_) ≠ −*α*_*cb*_(−H_a_) (Fig. 4f) is allowed^35^. Most importantly, a maximum |*α*_ANE_| of 10 A m^−1^ K^−1^ was achieved in the *bc* configuration, which is much higher than those of most of the ferromagnets, including Co_3_Sn_2_S_2_ (ref. ^7^), Co_2_MnGa (ref. ^9^), Fe_3_Ga (ref. ^2^) and SmCo_5_ (ref. ^36^; the record value for which is ~5 A m^−1^ K^−1^) and one order of magnitude higher than those of the antiferromagnets Mn_3_*X* (*X* = Sn (refs. ^6,12^) and Ge (refs. ^10,32^)).

There could be extrinsic contributions to such a large *α*_ANE_ in addition to the Berry curvature, particularly considering the following three aspects. First, there is a mismatch between the experimental results and those from first-principles predictions. The calculated |*α*_ANE_| is much lower than the experimental *α*_*bc*_, even when *E*_F_ was optimized (Supplementary Fig. 10). The negative temperature dependence of |*α*_ANE_| is similar to that recently been observed in MnBi, which was attributed to an extrinsic magnon-drag effect and could account for the deviation in temperature dependence between experiment and theory^37^. Second, anisotropic behaviour was observed in *α*_ANE_; that is, *α*_*bc*_ ≠ −*α*_*cb*_. In principle, from density functional theory, *α*_*bc*_^intrinsic^ = −*α*_*cb*_^intrinsic^ only if it solely originated from the Berry curvature, as determined by the Berry phase of the wavefunction, *Ω*_ij_(**k**) = −*Ω*_*ji*_(**k**). Third, a large violation of the ratio of |*α*_*bc*_/*σ*_*bc*_| from an empirical value of *k*_B_/*e* (where *k*_B_ is the Boltzmann constant) was observed (Supplementary Fig. 11). As recently argued by Ding et al. and Xu et al., the ratio of |*α*_ANE_/*σ*_ANE_| saturates to ~*k*_B_/*e* at 300 K if both originate from intrinsic Berry curvature^8,38^.

In fact, extrinsic contributions are possible in real crystals owing to the unavoidable presence of scattering—particularly for systems with large SOC, which is the case for YbMnBi_2_. The large SOC induces an additional skew force on the spin-polarized charge carriers^39,40^. Previous materials with large ANEs, including Co_3_Sn_2_S_2_^7^, Co_2_MnGa^9^, Fe_3_Ga^2^ and Mn_3_*X* (*X* = Sn and Ge)^6,10,12,32^, have small SOC without heavy elements, which is not the case here. Very recently, Papaj and Fu^41^ indicated that extrinsic contributions can be dramatic, and even potentially dominant over Berry curvature. To accurately describe *α*_ANE_, vast improvements to current modelling techniques, beyond the simple single band models, are necessary. Such improvements include, but are not limited to, the modelling of band anisotropy, deviations from a linear band dispersion and interactions of point defects with electrons^41^. The strong band anisotropy in YbMnBi_2_ has rarely been observed in other materials with large ANEs. We highlight that the strong anisotropy in the Fermi surface of YbMnBi_2_ plays a critical role in achieving large *α*_ANE_ in the *bc* configuration and not in the *cb* configuration. The electron pockets are highly dispersive in the *ab* plane but have nearly no dispersion along the *c* axis, leading to a considerable difference in the extrinsic scattering effects on charge carriers between the *bc* and *cb* configurations. The extremely high *σ*_*bb*_ (which is ~35 times higher than *σ*_*cc*_ at 80 K), along with asymmetric skew scattering rates, results in large *α*_ANE_ in only the *bc* configuration. Future investigations are warranted to explore both the intrinsic (for example, by developing a detailed distribution of the Berry curvature in the Brillouin zone) and extrinsic (for example, by measuring the strength of the skew scattering and side jump related to the SOC) contributions to the large *α*_ANE_ in more detail.

## Beyond the ANE

**Fig. 5:**
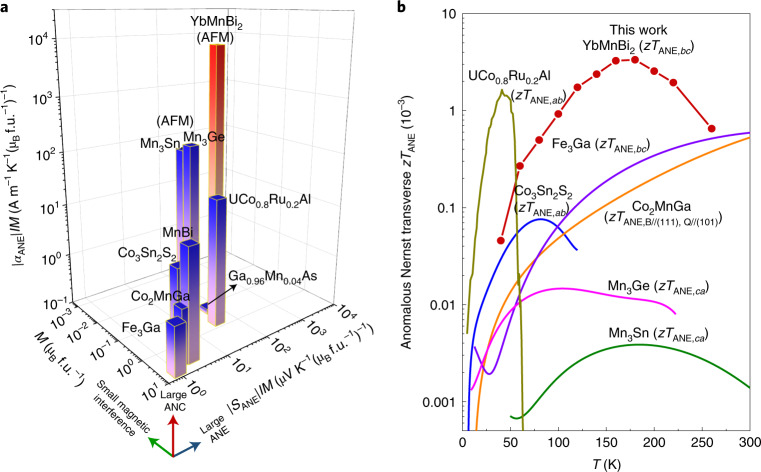
Thermoelectric performance comparison. **a**, Comparison of the absolute values of |*α*_ANE_|/*M*, |*S*_ANE_|/*M* and *M* of YbMnBi_2_ in the *bc* configuration (160 K) with those of other compounds with large ANE thermopowers—namely, the ferromagnets Fe_3_Ga (300 K)^2^, Co_2_MnGa (300 K)^3,9^, Co_3_Sn_2_S_2_ (80 K)^7^, UCo_0.8_Ru_0.2_Al (40 K)^15^, MnBi (80 K)^37^, Ga_1−*x*_Mn_*x*_As (10 K)^42^, and the chiral antiferromagnets Mn_3_Sn (300 K)^6,12^ and Mn_3_Ge (300 K)^10,32^. Antiferromagnets are marked as AFM, and other materials are all ferromagnets. **b**, The temperature dependence of *zT*_ANE_ values for YbMnBi_2_ in the *bc* configuration compared with those of other compounds with large ANE thermopowers.

In addition to the large ANE signals, YbMnBi_2_ presents extremely small magnetization *M*, which can reduce the interaction of the magnetic field with surrounding electronic devices during practical applications. Figure 5a compares |*α*_ANE_|/*M*, |*S*_ANE_|/*M* and *M* of YbMnBi_2_ with those of other compounds with large *S*_ANE_—namely, the ferromagnets Fe_3_Ga (ref. ^2^), Co_2_MnGa (refs.^3,9^), Co_3_Sn_2_S_2_ (ref. ^7^), UCo_0.8_Ru_0.2_Al (ref. ^15^), MnBi (ref. ^37^) and Ga_1−*x*_Mn_*x*_As (ref. ^42^) and the chiral antiferromagnets Mn_3_Sn (refs. ^6,12^) and Mn_3_Ge (refs. ^10,32^). The noncollinear antiferromagnets, especially YbMnBi_2_, clearly surpass all of the ferromagnets in terms of |*α*_ANE_|/*M* and |*S*_ANE_|/*M*, demonstrating that canted antiferromagnets, even with extremely low magnetization, can display a large ANE that competes with the best ferromagnets. For practical applications, using permanent magnets (with which fields of 0.3 T are common, and 1 T is possible with a careful choice of materials) is reasonable for materials with no remanent magnetization, such as YbMnBi_2_. Further exploration of the zero-field ANEs of polycrystals and/or thin films would be of interest for future devices.

In addition to the large ANE signals (owing to the anisotropy), the resistivity and thermal conductivity can be decoupled to realize simultaneous gains in both—that is, low *ρ*_*bb*_ and low *κ*_*cc*_ (*κ*_cc_ is the thermal conductivity along the *c* axis) (Supplementary Fig. 1). Both *ρ*_*bb*_ and *κ*_*cc*_ are much lower than the values for the ferromagnets with a large ANE (Supplementary Table 1). Consequently, YbMnBi_2_ shows a high anomalous Nernst thermoelectric figure of merit *zT*_ANE_, defined as, *zT*_ANE,*bc*_ = *S*_ANE,*bc*_^2^*T*/(*ρ*_*bb*_*κ*_*cc*_). As shown in Fig. 5b, YbMnBi_2_ has high *zT*_ANE_ values over a wide temperature range, demonstrating its highly promising anomalous transverse thermoelectric performance. Although the *zT*_ANE_ values are still much lower than the traditional, longitudinal *zT*, we present this result as promising progress towards the use of the ANE in thermoelectric applications, which could ultimately offer improvements in microscale devices and the field of spin caloritronics.

## Outlook

In summary, YbMnBi_2_, a canted antiferromagnet, breaks time-reversal symmetry and yields a non-zero Berry curvature. Alongside the large SOC contributed by the heavy Bi atoms, a large ANE conductivity of 10 A m^−1^ K^−1^ was observed, which is two to three times larger than those of most of the ferromagnets and compares favourably with those of noncollinear antiferromagnets. This is of great interest, particularly considering its small magnetization. Furthermore, it shows strong anisotropy, making it possible to achieve low resistivity and low thermal conductivity simultaneously in transverse thermoelectric configurations. All of these unique features demonstrate the great potential of YbMnBi_2_ as a transverse thermoelectric material.

These results suggest that canted antiferromagnets, including (but not limited to) the *R*MnPn_2_ family, are worthy of further investigation for large ANEs. Future studies searching for compounds with canting temperatures higher than room temperature are crucial. Moreover, investigating the use of polycrystalline or thin-film materials to induce remanent magnetizations could be important for practical applications without external magnetic fields. Precise tuning of the Fermi level would also be an efficient way to enhance the ANE. Finally, with a deeper understanding of the intrinsic and extrinsic contributions to the ANE, both experimentally and theoretically, we expect to achieve boosted ANEs in more topological materials.

## Methods

### Ab initio calculations

Calculations were conducted using density functional theory implemented in the Vienna ab initio simulation package (VASP) code^43,44^. To calculate the band structure, the Perdew–Burke–Ernzerhof exchange-correlation functional and projector-augmented-wave approach were used. The cut-off energy was set at 500 eV to expand the wavefunctions into a plane–wave basis. The Brillouin zone was sampled in the k-space within the Monkhorst–Pack scheme^45^, and a *k* mesh of 10 × 10 × 4 was adopted based on an equilibrium structure. The C-type antiferromagnetic order along the *c* axis at the Mn sites, a canted spin structure and SOC were considered. To calculate the anomalous Hall conductivity and anomalous Nernst conductivity, the ab initio density functional theory Bloch wavefunction was projected onto highly symmetric atomic-orbital-like Wannier functions^46^ with a diagonal position operator using VASP code^43,44^. To obtain precise Wannier functions, we included the outermost *s* and *d* orbitals for Yb, *d* orbital for Mn and *p* orbital for Bi to cover the full band overlap from the ab initio and Wannier functions. The atomic coordinates for the electronic structure calculations are shown in Supplementary Table 2.

### Sample preparation

YbMnBi_2_ single crystals were grown using a self-flux method with an elemental ratio of Yb:Mn:Bi of 1:1:4. Yb (99.99%), Mn (99.98%) and Bi (99.999%) were cut into small pieces and mixed before being placed in an alumina crucible. They were then sealed in a quartz tube under a partial argon pressure. The sealed tube was heated to 1,050 °C over 2.5 days and maintained at that tempearture for 24 h. Next, it was slowly cooled to 400 °C at a rate of 2 °C h^−1^, and single crystals were obtained by removing the flux through centrifugation.

### Sample characterization

The single crystallinity and orientation of the as-grown single crystals were determined using Laue X-ray diffraction (Supplementary Fig. 12). The composition and homogeneity were examined by scanning electron microscopy (Philips XL30) with an Oxford energy-dispersive X-ray spectroscopy (Quantax, Bruker) (Supplementary Fig. 13).

### ARPES measurements

The ARPES experiment was performed at the Bloch beamline at MAX IV with a Scienta DA30 analyser. The sample was cleaved in situ at 19 K with a base pressure lower than 1 × 10^−10^ mbar.

### Measurement of transport properties

Resistivities and Hall resistivities were measured using a physical property measurement system (PPMS9, Quantum Design) in an electrical transport option via a standard four-probe method. The Nernst thermopower, Seebeck coefficient and thermal conductivity were measured in the PPMS9 under a high vacuum by using a standard four-contact steady-state method^47^. The magnetization was measured using a magnetic property measurement system (MPMS3, Quantum Design).

## Online content

Any methods, additional references, Nature Research reporting summaries, source data, extended data, supplementary information, acknowledgements, peer review information; details of author contributions and competing interests; and statements of data and code availability are available at 10.1038/s41563-021-01149-2.

## Supplementary information

Supplementary Information Supplementary Figs. 1–13, Tables 1 and 2, Discussion and refs. 1–16.

## Data Availability

All the data supporting the plots within this paper and the findings of this study are available from the corresponding author upon request.
