# Supplementary material for: Giant anomalous Nernst signal in the antiferromagnet YbMnBi2
Source: Nat Mater. 2021 Nov 22;21(2):203–9. doi: 10.1038/s41563-021-01149-2 (PMC8810386; doi:10.1038/s41563-021-01149-2)
Supplement: Supplementary file 1 — Supplementary Figs. 1–13, Tables 1 and 2, Discussion and refs. 1–16. [file 41563_2021_1149_MOESM1_ESM.pdf]

nature portfolio

https://doi.org/10.1038/s41563-021-01149-2

Supplementary information

Giant anomalous Nernst signal in the antiferromagnet $YbMnBi_2$

In the format provided by the authors and unedited

Supporting Information

Giant anomalous Nernst signal in the antiferromagnet $YbMnBi_2$

Yu Pan[1,*], Congcong Le[1], Bin He[1], Sarah J. Watzman[1,2,3], Mengyu Yao[1], Johannes Gooth[1], Joseph P. Heremans[2,4,5], Yan Sun[1], Claudia Felser[1,*]

[1]*Max Planck Institute for Chemical Physics of Solids, Nöthnitzer Str. 40, Dresden 01187, Germany.*

[2]*Department of Mechanical and Aerospace Engineering, The Ohio State University, Columbus, OH 43210, USA.*

[3]*Department of Mechanical and Materials Engineering, University of Cincinnati, Cincinnati, OH 45221, USA.*

[4]*Department of Materials Science and Engineering, The Ohio State University, Columbus, OH 43210, USA.*

[5]*Department of Physics, The Ohio State University, Columbus, OH 43210, USA.*

E-mail address: Yu.Pan@cpfs.mpg.de; Claudia.Felser@cpfs.mpg.de

Temperature dependence of resistivity and thermal conductivity

As shown in **Fig. S1**(a), a clear Néel temperature T_N is observed in the temperature dependent resistivity. In addition, strong anisotropy is observed in both resistivity and thermal conductivity. The resistivity in the *ab*-plane is much lower than that along the *c*-axis. Phonons also show a slower velocity along the *c*-axis than in the *ab*-plane, because of which the thermal conductivity along the *c*-axis is much lower than that in the *ab*-plane (**Fig. S1**(b)). Because of such large anisotropy, a low resistivity and low thermal conductivity can simultaneously be achieved for certain transverse thermoelectric configurations. For example, by applying the *bc* configuration for ANE applications, a high anomalous transverse figure of merit $zT_{bc} = S_{bc}^2T/\rho_{bb}\kappa_{cc}$ can be achieved due to simultaneous gain in resistivity and thermal conductivity, *i.e.* low ρ_{bb} and extremely low κ_{cc}.

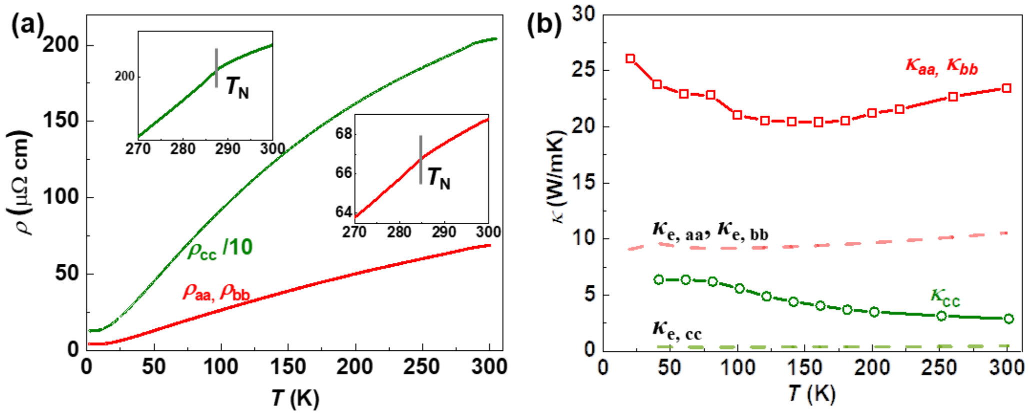

Fig. S1 Temperature dependence of (a) resistivity and (b) thermal conductivity in the *ab*-plane and *c*-axis. The insets of (a) are magnified views showing the Néel temperature T_N.

Magnetism measurement

The magnetic field dependence of the magnetization curves indicates a canted antiferromagnetic feature of $YbMnBi_2$. Along *c*-axis, a saturation is observed at low temperatures at ~4-6 T (**Fig. S2**(a)), demonstrating the non-collinear antiferromagnetic structure of $YbMnBi_2$. Additionally, since it is extremely hard to push all the spin in the *ab*-

plane, no saturation is observed until 7 T in the *ab*-plane, as shown in **Fig. S2**(b). **Fig. S2**(c)-(d) shows the hysteresis taking 160 K as an example.

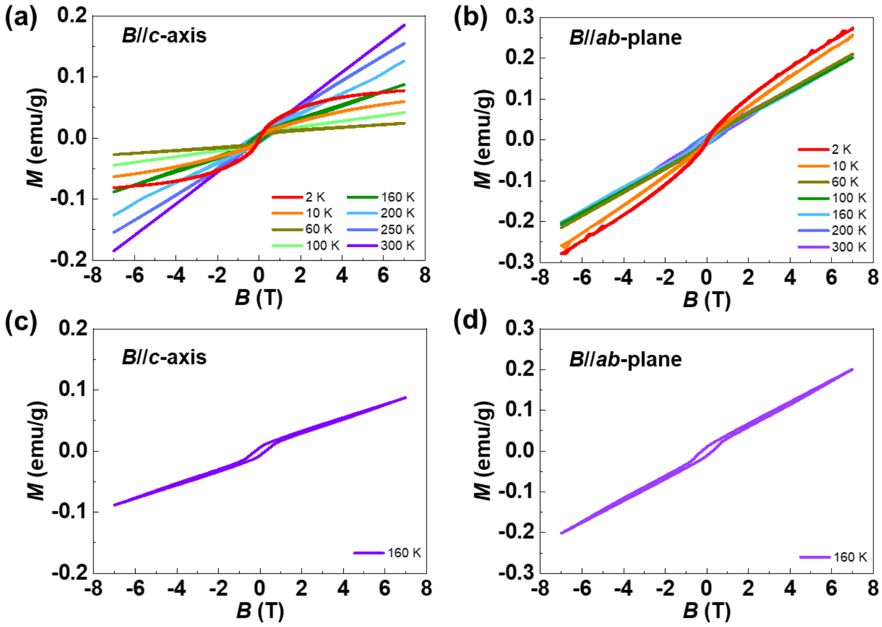

Fig. S2 Magnetic field dependence of magnetization: (a) and (c) *B* parallel to *c*-axis, (b) and (d) *B* parallel to *ab*-plane.

<u>ANE/AHE measurements</u>

For the measurements of thermal transport properties, the sample was fixed on a piece of alumina heater sink, with a strain gauge heater attached to the other end to apply a temperature gradient. Two sets of chromel-constantan thermocouples were mounted at two points along the temperature gradient to measure the temperature difference, and chromel thermocouples were used to measure the voltage difference.

Anomalous Nernst/Hall effects shows up when the magnetic field is along *a* // (100), while no anomalous Nernst thermopower (**Fig. S3**) or Hall signal (**Fig. S4**) is observed when the magnetic field is along *c* // (001). These results are highly consistent with the theoretical predication, indicating that the origin of the ANE is the intrinsic Berry curvature.

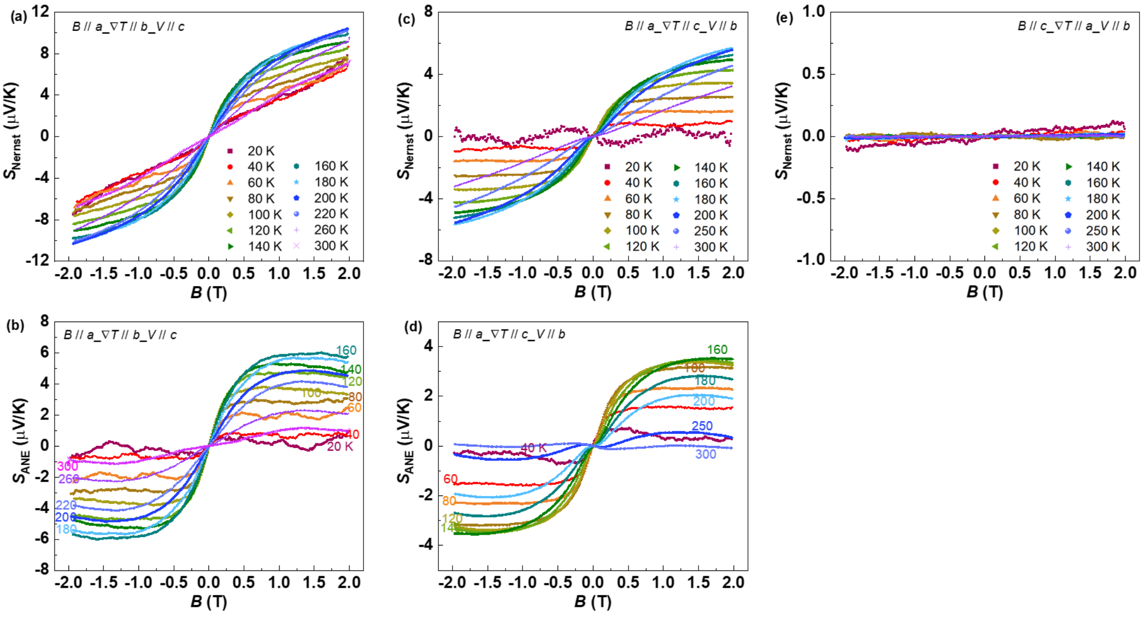

Fig. S3 Magnetic field dependence of Nernst thermopower (a), (c) (e) and analyzed anomalous Nernst signal (b), (d) along different directions: (a), (b) *B* along *a* // (100), temperature gradient along *b* // (010), Nernst voltage along *c* // (001); (c), (d) *B* along *a* // (100), temperature gradient along *c* // (001), Nernst voltage along *b* // (010); (e) *B* along *c* // (001), temperature gradient along *a* // (100), Nernst voltage along *b* // (010), respectively.

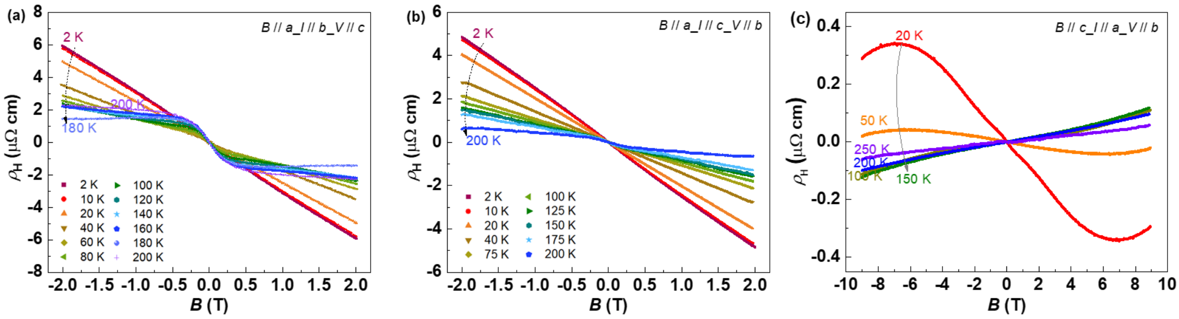

Fig. S4 Magnetic field dependence of Hall resistivity along different directions: (a) *B* along *a* // (100), current along *b* // (010) and Hall voltage along *c* // (001), (b) *B* along *a* // (100), current along *c* // (001) and Hall voltage along *b* // (010), (c) *B* along *c* // (001), current along *a* // (100) and Hall voltage along *b* // (010).

On the other hand, at low temperatures, the Hall resistivity is non-linear change from negative to positive from low to high magnetic field (**Fig. S4**(c)), indicative of a two-types of charge carrier behavior, and electrons dominate at low fields. More importantly, the slope of the Hall resistivity goes through a negative to positive change, indicating a n- to p-type transition between 50-100 K.

Sign convention of Nernst and Hall

Fig. S5 illustrates the sign convention of Nernst and Hall measurements.

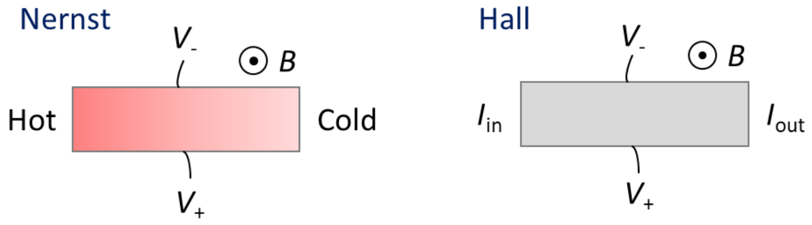

Fig. S5 Sign convention of Nernst and Hall measurements.

The Gerlach sign convention of the measured Nernst thermopower and Hall resistivity are shown in **Fig. S5**. As shown in **Fig. S5**(a), in the condition with a heater on the left side (hot) and a heat sink on the right side (cold), and a magnetic field *B* out of paper, the bottom side of the sample is wired to the positive voltage (V_+) and the upper side of the sample is wired to the negative voltage (V_-) of the voltmeter. Similarly, as shown in **Fig. S5**(a), in the condition with a current input (I_{in}) on the left side and a current output (I_{out}) on the right side, and a magnetic field *B* out of paper, the bottom side of the sample is wired to the positive voltage (V_+) and the upper side of the sample is wired to the negative voltage (V_-) of the voltmeter.

DFT calculated band structure

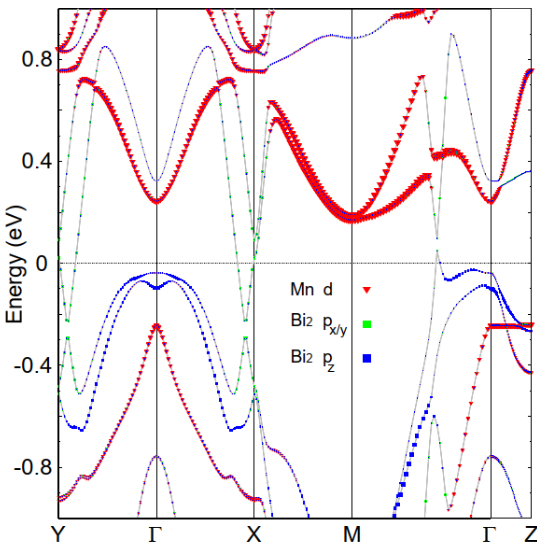

Fig. S6 First principle calculated band structure by assuming a canting angle of zero.

Assuming a canting angle of zero, the band structure shows that there is no contribution from the hole pocket at Γ point, as shown in **Fig. S6**. By varying the canting angle from 8 º to 18 º, it is found that the valence band at Γ point contribute more and more to the conduction, as shown in **Fig. S7**. Topological bands contributed by the *p* orbitals of Bi hardly changes with canting angle, but a larger canting angle results in an obvious change in the bands at Γ point since they are contributed by the hybridization of the p_z orbitals of Bi and the *d* orbitals of Mn. Most importantly, the joint of the bands at Γ point results in a larger positive Berry curvature, which is advantageous for stronger ANE/AHE signals.

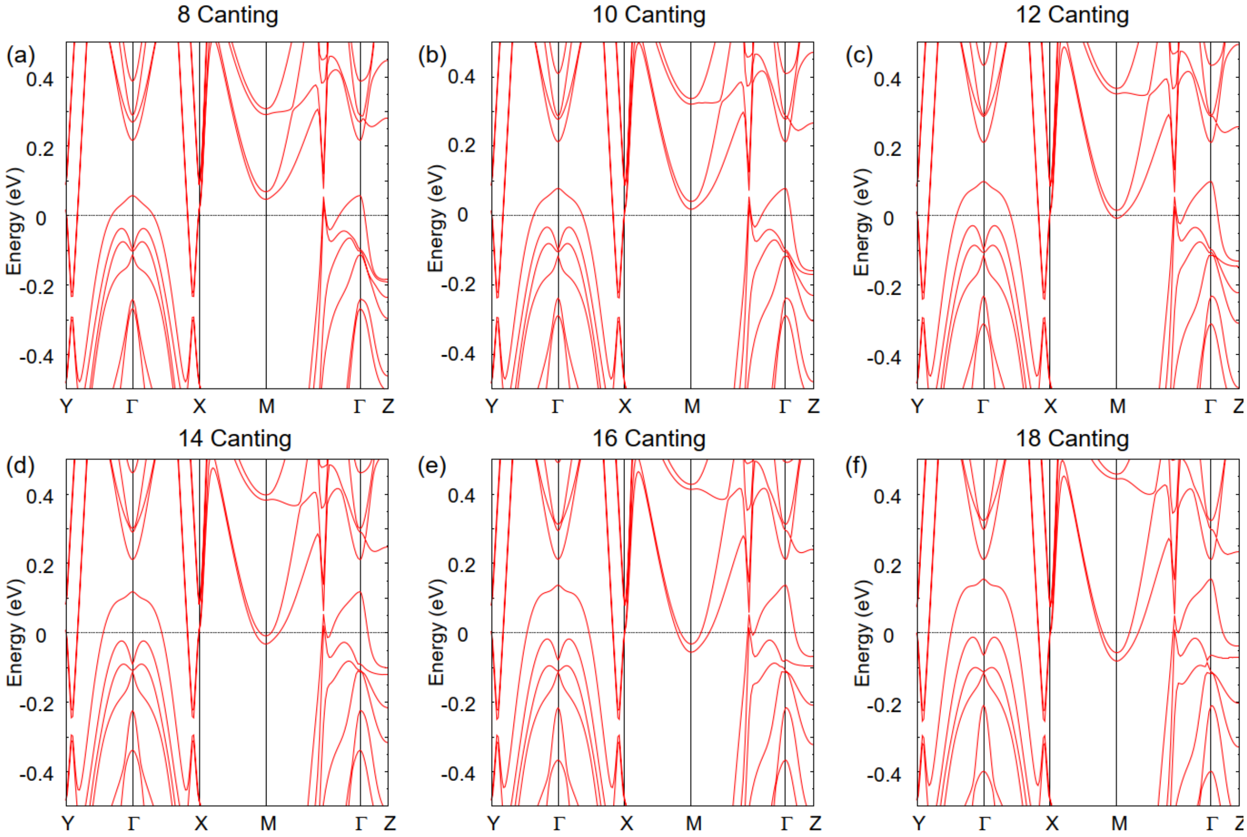

Fig. S7 First principle calculated band structure with different canting angles.

MR measurement

Magnetoresistance along different directions are shown in **Fig. S8**. Maximum MR is obtained when *B* is along *c* // (001) and current is along *a* // (100), as shown in **Fig. S8**(a). Because of this sharp band dispersion, the *ab*-plane resistivity of our high quality $YbMnBi_2$ single crystal and undergoes clear Shubnikov-de Haas (S-dH) oscillations at $B > 5$ T from 2 K to 10 K (**Fig. S8**(a)). By subtracting a smooth background, **Fig. S8**(d) illustrates the oscillation amplitudes of resistivity against 1/*B*, which shrinks with increasing temperature. **Fig. S8**(e) show that the temperature dependence of the oscillation amplitudes can be well fitted by the Lifshitz-Kosevich (LK) formula,[1] which denotes an effective mass $m*$ of ~0.23 m_0. This value is close to a previous study [2] and it is quite small compared to ferromagnets. **Fig. S8**(f) schematically show that the sharp dispersion of the topological bands in *ab*-plane, while show almost no dispersion in *c*-axis. That's why oscillation is only observed when the magnetic field is along (001).

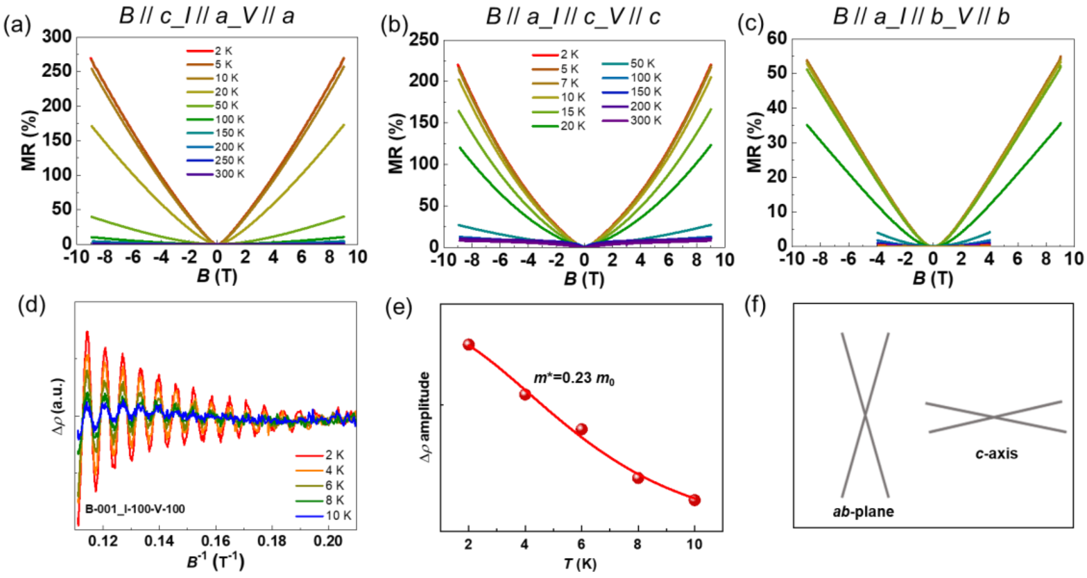

Fig. S8 Magneto resistance along different directions: (a) magnetic field *B* along *c* // (001), current *I* along *a* // (100); (b) *B* along *a* // (100), *I* along *c* // (001); and (c) *B* along *a* // (100), *I* along *b* // (010). (d)-(e) analyzed the oscillation of MR in (a) below 10 K and resolve an effective mass $m*$ of 0.23 m_0. (f) A schematic illustration of the highly anisotropic topological bands.

Seebeck coefficient

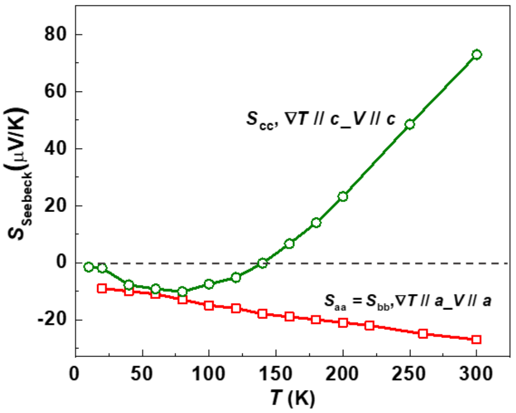

Fig. S9 Temperature dependence of the Seebeck coefficient along *a*-axis (100) S_{aa} and *c*-axis (001) S_{cc}. $S_{aa} = S_{bb}$ owing to the tetragonal crystal structure.

Seebeck coefficient along different directions are shown in **Fig. S9**. The turn up of Seebeck coefficient along c-axis happened at 50-100 K, which is also because of the shift of Fermi level, similar to Hall but more complicated as it depends on not only the conductivity but also the Seebeck coefficient of the two types of charge carriers. It is known that for semimetals, $S_{Seebeck,i} = (S_{e,i}\sigma_{e,i} + S_{h,i}\sigma_{h,i})/(\sigma_{e,i} + \sigma_{h,i})$, [3] in which S_e and S_h is the Seebeck coefficient of the electrons and holes, and the subscribe i represents ab plane or c-axis. Hence the total Seebeck coefficient depends on the competition between $S_e\sigma_{e,i}$ and $S_h\sigma_{h,i}$ (note S_h is always positive while S_e is always negative). At low temperatures where electrons dominate the conduction, $|S_e\sigma_{e,i}|$ would always larger than $S_h\sigma_{h,i}$, leading to negative Seebeck coefficient. While at higher temperatures, $S_h\sigma_{h,i}$ can be larger than $|S_e|\sigma_{e,i}$ with more holes join in the conduction, which is particularly effective in the c-axis as $\sigma_{e,c}$ is very low, again due to the extremely flat dispersion of the topological bands along c-axis. This results further demonstrate the significant shift of Fermi level with temperature.

On the other hand, co-exist of positive and negative Seebeck coefficient in different crystallographic directions are observed, which may indicate that $YbMnBi_2$ is a goniopolar material.[4]

Anomalous Hall/Nernst conductivity

Experimentally, σ_{AHE} (taking cb as an example) is calculated by:

$$\sigma_{cb} = \frac{-\rho_{cb}}{\rho_{bb}\rho_{cc} - \rho_{bc}\rho_{cb}} \approx \frac{-\rho_{cb}}{\rho_{bb}\rho_{cc}}, \quad (1)$$

where $\rho_{bb}\rho_{cc} \gg \rho_{bc}\rho_{cb}$. The anomalous Nernst conductivity α_{ANE} in cb (V // c) is calculated as:

$$\alpha_{cb} = S_{cb}\sigma_{cc} + S_{bb}\sigma_{cb}, \quad (2)$$

where S_{cb} and S_{bb} are the S_{ANE} in cb and Seebeck coefficient along the b-axis, respectively. In our results, because $S_{cb}\sigma_{cc} \gg S_{bb}\sigma_{cb}$, $S_{cb}\sigma_{cc}$ makes the dominant contribution to α_{ANE}. Because of the extremely high σ_{bb} (~35 times higher than σ_{cc} at 80 K) and comparable S_{bc}, high α_{ANE} is achieved in bc rather than cb. Such large difference between σ_{bb} and σ_{cc} originates from the strong anisotropy of the Fermi surface, in which the electron pockets are highly dispersive in ab-plane but have nearly no dispersion along c-axis.

Fig. S10 shows the first-principles calculated AHE conductivity as a function of Fermi energy and ANE conductivity as a function of temperature at different canting angles. The calculated ANE conductivity increases with temperature up to 250 K. The absolute values of the ANE conductivity show no obvious change by varying the canting angle or shift the Fermi energy.

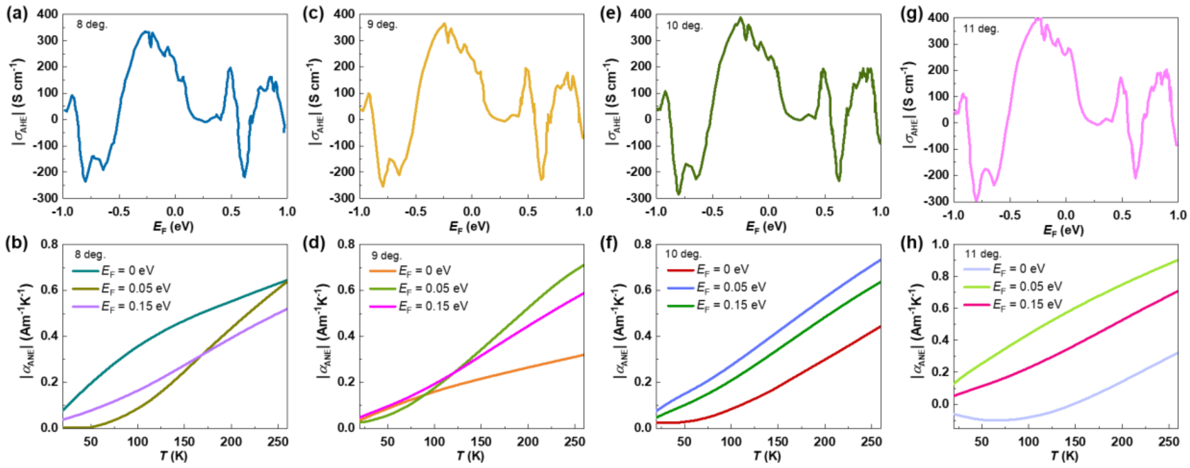

Fig. S10 First-principles calculated AHE conductivity as a function of Fermi energy and ANE conductivity as a function of temperature at different canting angles: (a), (b) 8 degrees, (c), (d) 9 degrees, (e), (f) 10 degrees, (g), (h) 11 degrees.

The ratio of anomalous Nernst conductivity to anomalous Hall conductivity

As argued by Behnia *et al.* recently,[5] the ratio of anomalous Nernst conductivity (α_{ij}) to anomalous Hall conductivity (σ_{ij}) would reach ~k_B/e at 300 K if both AHE and ANE are dominated by the intrinsic Berry curvature contribution. Therefore, we analyzed the scaling ratio of the α_{ij}/σ_{ij} of $YbMnBi_2$, as shown below in **Fig. S11**. It is found that the ratio in *cb* (α_{cb}/σ_{cb}) is within a few k_B/e, however, there is a surprisingly large violation from k_B/e in *bc* (α_{bc}/σ_{bc}). The reasons for the large violation can be probably understood from four aspects as discussed below.

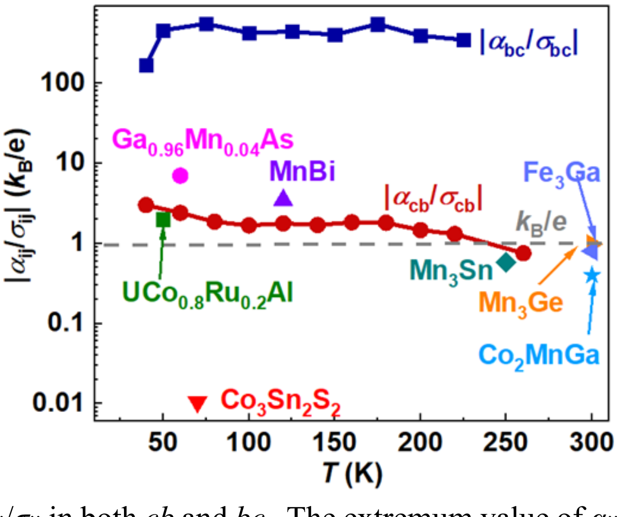

Fig. S11 The ratio of α_{ij}/σ_{ij} in both *cb* and *bc*. The extremum value of α_{ij}/σ_{ij} in other compounds are shown to compare, including Co_2MnGa (300 K), [5] $Co_3Sn_2S_2$ (70 K), [5] $Ga_{0.96}Mn_{0.04}As$ (60 K), [6] $UCo_{0.8}Ru_{0.2}Al$ (50 K), [7] Mn_3Sn (250 K), [5] Mn_3Ge (300 K), [5] Fe_3Ga (300 K), [8] and MnBi (120 K). [9]

First, there can be an extrinsic contribution to AHE and ANE in $YbMnBi_2$, so that the AHE and ANE conductivities are not simply determined by the Berry curvature. Second, since the empirical observation of ~k_B/e in the model is constructed based on a two-dimensional circular isotropic Fermi surface,[5,10] it is reasonable that $YbMnBi_2$ is an exception since its Fermi surface are highly anisotropic (highly dispersive in *ab*-plane but presents almost no dispersion along *c*-axis). Moreover, the topological band in $YbMnBi_2$ is highly linear, which is very

different from a parabolic band as the model requires.[10] A precise description of the Fermi surface in the 3-dimensional Brillouin zone by considering the particular conductions, for example, the anisotropy of the band structure, the deviations of the band dispersion from parabolic spectrum, *etc.* is essential in the case of $YbMnBi_2$. In $YbMnBi_2$, $|\alpha_{ij}/\sigma_{ij}| \approx |S_{ij}*\sigma_{ii}|/|(\rho_{ij}/\rho_{ii}\rho_{jj})| = S_{ij}*\rho_{jj}/\rho_{ij}$. Since the ratio of the resistivity between ab-plane and c-axis is very large, while both S_{ij} and ρ_{ij} are almost at the same order in ab-plane and c-axis, the large difference in $|\alpha_{ij}/\sigma_{ij}|$ originates from the significant difference of ρ_{bb} and ρ_{cc}. In fact, for most of the materials with large ANE observed so far, a large difference in ρ_{ii} and ρ_{jj} has never been observed, even including Mn_3Sn and Mn_3Ge. Third, it maybe also because that there are more than one band so that the single-band approach on longer stands. At last, the empirical ration of $\sim k_B/e$ is a high temperature limit near 300 K, which $YbMnBi_2$ may never reach since its canting temperature is ~250 K. As can be seen in **Fig. S11**, most of the materials approach the empirical value of $\sim k_B/e$ at ~300 K, while there can be a violation at low temperatures below 300 K, for example, $Ga_{0.96}Mn_{0.04}As$ (60 K), MnBi (120 K), $UCo_{0.8}Ru_{0.2}Al$ (50 K) show a ratio of few $\sim k_B/e$, and $Co_3Sn_2S_2$ (70 K) shows a ratio two orders lower than $\sim k_B/e$ (only ~0.01 $\sim k_B/e$).

Single crystal characterization

Laue X-ray diffraction pattern of the sample demonstrates the high quality of the single crystal, as shown by the distinct spots in **Fig. S12**. Additionally, the single crystals tend to grow in a layered structured with the surface being *ab*-plane.

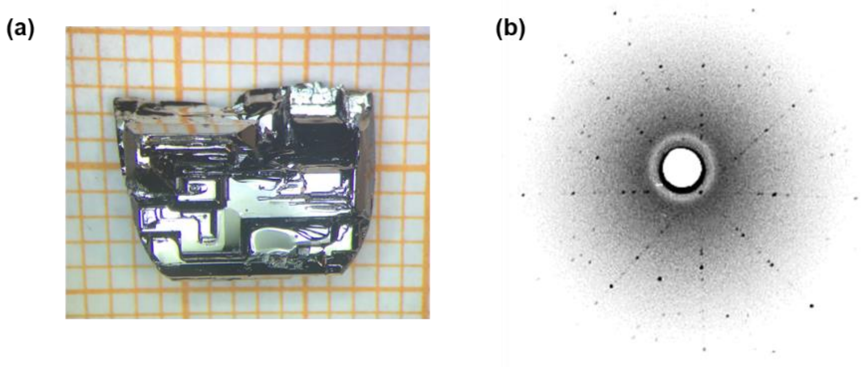

Fig. S12 (a) Optical image of the as-grown single crystal. (b) Laue pattern of $YbMnBi_2$ single crystal.

The backscattered electrons (BSE) image and Yb, Mn, Bi mapping confirm the uniform distribution of the elements (**Fig. S13**(a)). Further energy-dispersive X-ray spectroscopy (EDX) analysis (**Fig. S13**(b)) indicates the composition ratio of Yb:Mn:Bi as 25.6:24.9:49.5, which agrees well with the stoichiometric 1:1:2 ratio.

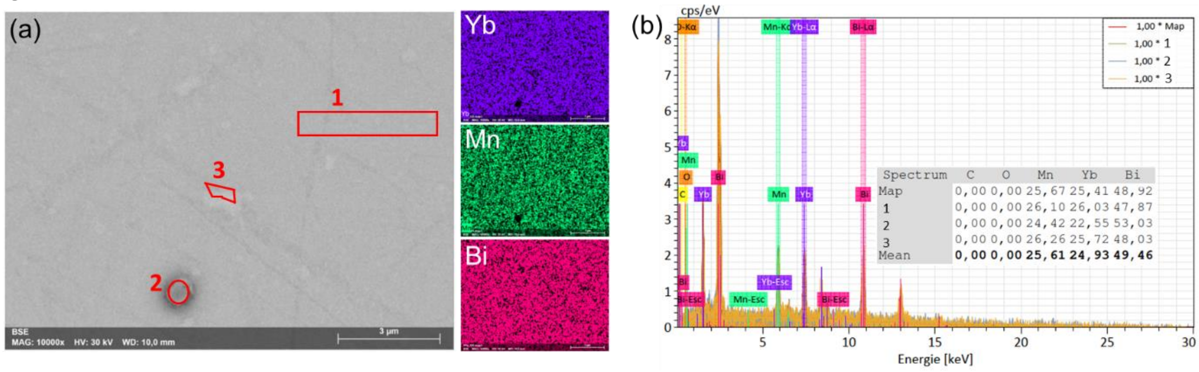

Fig. S13 (a) Backscattered electrons (BSE) image and Yb, Mn, Bi mapping, (b) Energy-

dispersive X-ray spectroscopy (EDX) analysis of $YbMnBi_2$ single crystal.

With a comparable ANE thermopower of few microvolts per Kelvin, in the *bc* configuration, $YbMnBi_2$ shows a lower resistivity and much lower thermal conductivity than other compounds, as shown in **Table S1**.

Table S1 Resistivity and thermal conductivity of $YbMnBi_2$, with a comparison to other ferromagnets with high ANE performance. Since different materials show the highest ANE performance at different temperatures, values at 300 K are adopted here for a rough comparison.

Compound	Resistivity ρ	Thermal conductivity κ
$YbMnBi_2$	ρ_{bb} = 68 μΩ cm	κ_{cc} = 2.88 $Wm^{-1}K^{-1}$
$Co_3Sn_2S_2$[11]	ρ_{aa} ~ 380 μΩ cm	κ_{bb} ~ 3.5 $Wm^{-1}K^{-1}$
Co_2MnGa[12,13]	ρ ~ 120 μΩ cm	κ ~ 22 $Wm^{-1}K^{-1}$
Fe_3Ga[8]	ρ ~ 85 μΩ cm	κ ~ 20 $Wm^{-1}K^{-1}$
Mn_3Sn[14,15]	ρ_{cc} ~ 300 μΩ cm	κ_{aa} ~ 8 $Wm^{-1}K^{-1}$
Mn_3Ge[16]	ρ_{cc} ~ 175 μΩ cm	κ_{aa} ~ 10 $Wm^{-1}K^{-1}$ (200 K)

Table S2 Atomic coordinates for the electronic structure calculations.

a=b=4.461Å c=10.732Å Symmetry group: P4/nmm			
Atom	x	y	z
Yb	0.0	0.5	0.731430
Yb	0.5	0.0	-0.731430
Mn	0.0	0.0	0.0
Mn	0.5	0.5	0.0
Bi	0.0	0.5	0.165670
Bi	0.5	0.0	-0.165670
Bi	0.0	0.0	0.5
Bi	0.5	0.5	0.5

References

1. Shoenberg, D. Magnetic Oscillations in Metals. Cambridge University Press, Cambridge, England, (1984).

2. Liu, J. Y. et al. Unusual interlayer quantum transport behavior caused by the zeroth Landau level in $YbMnBi_2$. *Nat. Commun.* **8**, 646 (2017).

3. Rowe, D. M. CRC Handbook of thermoelectics. CRC Press, Boca Raton, FL, USA (1995).

4. He, B. et al. The Fermi surface geometrical origin of axis-dependent conduction polarity in layered materials. *Nat. Mater.* **18**, 568 (2019).

5. Xu, L. et al. Anomalous transverse response of Co2MnGa and universality of the room-temperature $\alpha_{ij}^A/\sigma_{ij}^A$ ratio across topological magnets. *Phys. Rev. B* **101**, 180404(R) (2020).

6. Pu, Y. et al. Mott relation for anomalous Hall and Nernst Effects in Ga1-xMnxAs ferromagnetic semiconductors. *Phys. Rev. Lett.* **101**, 117208 (2008).

7. Asaba, T. et al. Colossal anomalous Nernst effect in a correlated noncentrosymmetric kagome

ferromagnet. *Sci. Adv.* **7**, eabf1467 (2021).

8. Sakai, A. et al. Iron-based binary ferromagnets for transverse thermoelectric conversion. *Nature* **581**, 53 (2020).
9. He, B. et al. Magnon-induced giant anomalous Nernst effect in single crystal MnBi. arXiv:2009.02211 (2020).
10. Ding, L. et al. Intrinsic anomalous Nernst effect amplified by disorder in a half-metallic semimetal. *Phys. Rev. X* **9**, 041061 (2019).
11. Guin, S. N. et al. Zero-field Nernst effect in a ferromagnetic kagome-lattice Weyl-semimetal Co3Sn2S2. *Adv. Mater.* **31**, 1806622 (2019).
12. Sakai, A. et al. Giant anomalous Nernst effect and quantum-critical scaling in a ferromagnetic semimetal. *Nat. Phys.* **14**, 1119 (2018).
13. Guin, S. N. et al. Anomalous Nernst effect beyond the magnetization scaling relation in the ferromagnetic Heusler compound Co2MnGa. *NPG Asia Mater.* **11**, 16 (2019).
14. Ikhlas, M. et al. Large anomalous Nernst effect at room temperature in a chiral antiferromagnet. *Nat. Phys.* **13**, 1085 (2017).
15. Tomita, T., Ikhlas, M. & Nakatsuji, S. Large Nernst effect and thermodynamics properties in Weyl antiferromagnet. *JPS Conf. Proc.* **30**, 011009 (2020).
16. Wuttke, C. et al. Berry curvature unravelled by the anomalous Nernst effect in Mn3Ge. *Phys. Rev. B* **100**, 085111 (2019).
